# Deletion of Gpr55 Results in Subtle Effects on Energy Metabolism, Motor Activity and Thermal Pain Sensation

**DOI:** 10.1371/journal.pone.0167965

**Published:** 2016-12-12

**Authors:** Mikael Bjursell, Erik Ryberg, Tingting Wu, Peter J. Greasley, Mohammad Bohlooly-Y, Stephan Hjorth

**Affiliations:** 1 Discovery Sciences Transgenics, AstraZeneca R&D, Mölndal, Sweden; 2 Cardiovascular and Metabolic diseases (CVMD) Innovative Medicines and early Development Biotech Unit, AstraZeneca R&D, Mölndal, Sweden; 3 CVMD Translational Medicine Unit, Early Clinical Development AstraZeneca R&D, Mölndal, Sweden; 4 Dept. of Molecular & Clinical Medicine, Inst. of Medicine, The Sahlgrenska Academy at Gothenburg University, Gothenburg, Sweden; Hospital Infantil Universitario Nino Jesus, SPAIN

## Abstract

The G-protein coupled receptor 55 (GPR55) is activated by cannabinoids and non-cannabinoid molecules and has been speculated to play a modulatory role in a large variety of physiological and pathological processes, including in metabolically perturbed states. We therefore generated male mice deficient in the gene coding for the cannabinoid/lysophosphatidylinositol (LPI) receptor *Gpr55* and characterized them under normal dietary conditions as well as during high energy dense diet feeding followed by challenge with the CB1 receptor antagonist/GPR55 agonist rimonabant. *Gpr55* deficient male mice (*Gpr55* KO) were phenotypically indistinguishable from their wild type (WT) siblings for the most part. However, *Gpr55* KO animals displayed an intriguing nocturnal pattern of motor activity and energy expenditure (EE). During the initial 6 hours of the night, motor activity was significantly elevated without any significant effect observed in EE. Interestingly, during the last 6 hours of the night motor activity was similar but EE was significantly decreased in the *Gpr55* KO mice. No significant difference in motor activity was detected during daytime, but EE was lower in the *Gpr55* KO compared to WT mice. The aforementioned patterns were not associated with alterations in energy intake, daytime core body temperature, body weight (BW) or composition, although a non-significant tendency to increased adiposity was seen in *Gpr55* KO compared to WT mice. Detailed analyses of daytime activity in the Open Field paradigm unveiled lower horizontal activity and rearing time for the *Gpr55* KO mice. Moreover, the *Gpr55* KO mice displayed significantly faster reaction time in the tail flick test, indicative of thermal hyperalgesia. The BW-decreasing effect of rimonabant in mice on long-term cafeteria diet did not differ between *Gpr55* KO and WT mice. In conclusion, *Gpr55* deficiency is associated with subtle effects on diurnal/nocturnal EE and motor activity behaviours but does not appear *per se* critically required for overall metabolism or behaviours.

## Introduction

Cannabinoids affect a large variety of physiological processes, including energy metabolism, appetite regulation, emesis and pain. Various cannabinoid molecules have been developed with aims to treat obesity, anorexia, neuralgia, inflammation, Parkinson’s and Alzheimer’s disease, epilepsy, cardiovascular disorders and cancer (reviewed in [1]). These multiple putative clinical indications suggest the existence of comprehensive and complex signalling systems for endogenous cannabinoid molecules (endocannabinoids) in peripheral tissues as well as in the central nervous system (CNS). GPCR′s identified that are activated by endocannabinoids, include the cannabinoid receptor 1 (CB1) [2, 3], 2 (CB2) [4], TRPV1 [5], GPR18 [6] GPR119 [7] and GPR55 [8].

*Gpr55* is expressed in various tissues, including white adipose tissue, liver, adrenal glands, gastrointestinal tract, as well as in the hypothalamus, frontal cortex and other CNS structures [8–11]. The expression pattern together with previous literature supports a role for GPR55 in the regulation of components involved in the control of energy homeostasis, pain, motor function and behaviour [12–14].

Lysophophatidylinositol (LPI) is reported to be the main endogenous ligand for GPR55 [15], and other putative endogenous GPR55 ligands are also active on other receptors and channels in similar concentrations [16, 17]. Due to the lack of robust and specific GPR55 assays, there are no well-characterized ligands for this target. Hence, the biological role of GPR55 is not entirely clear. *Gpr55* deficient mice have been developed and studied to understand the importance of the receptor in a variety of areas [18–21]. Wu *et al*. [14] reported that *Gpr55* deficient mice do not display any gross abnormalities, and no significant effects were observed on body weight, grip strength nor on behaviour in models used to detect anxiolytic- or antidepressant-like action. However, these mice displayed a trend towards decreased horizontal activity in the open field test, and significantly shorter reaction time in the hot plate test, indicating thermal hyperalgesia [14]. Another recent study reported that *Gpr55* deficient mice have unaltered body weight but when older (5–9 months) display increased fat mass and insulin resistance [12]. Moreover, this study also found that 5 months old *Gpr55* deficient mice had unaltered energy intake and respiratory exchange ratio, but reduced energy expenditure and motor activity. Also, a number of other recent reports have pointed to a putative role of *Gpr55* in metabolic disorder (see, e.g., [11, 22]).

In the present study, a new independent *Gpr55* deficient mouse line was developed and studied to understand the importance of GPR55 in metabolism and behaviours in young animals under normal dietary conditions. In addition, separate cohorts of *Gpr55* deficient mice were subjected to a longitudinal study during which high energy dense diets was provided followed by challenge with the CB1 receptor /GPR55 ligand rimonabant.

## Methods

### Generation of *Gpr55* deficient mice

**Fig 1:**
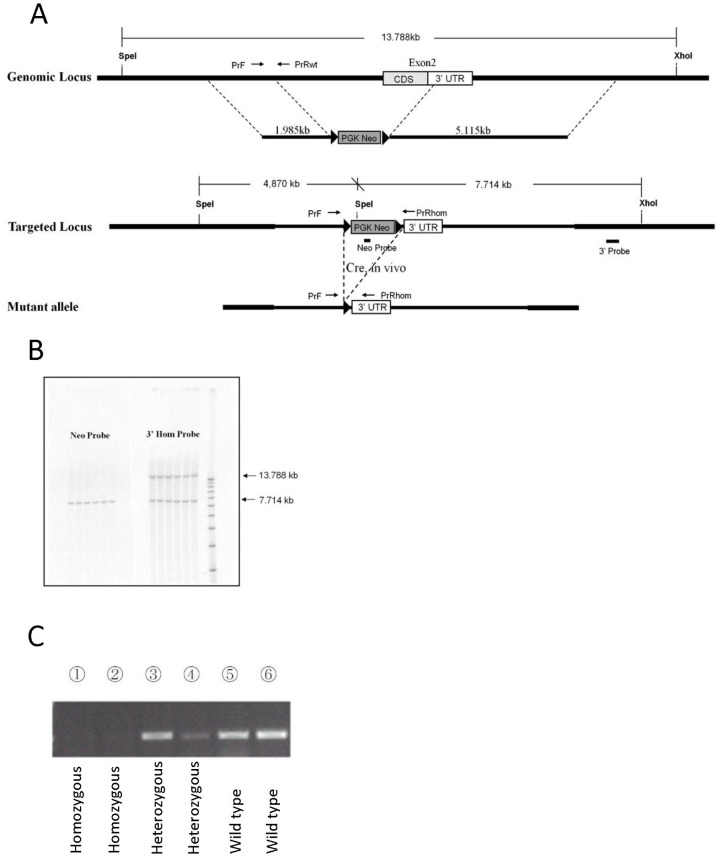
Construction and confirmation of *Gpr55* KO mice. (**A**) Schematic diagram of the mouse *Gpr55* gene and the targeting strategy. CDS: coding sequence, 3′UTR: 3 end un-translated region, PGK Neo: a loxP-flanked Neo cassette. (**B**) Southern blotting analyses of ES-cell clones using the probes indicated in (A). (**C**) *Gpr55* expression analyses by reverse transcriptase (RT) PCR analysis of cerebrum dissected from *Gpr55* homozygous KO (lane 1–2), *Gpr55* heterozygous (lane 3–4) and wild type (lane 5–6) mice.

For targeting vector construction, a 1.98kb 5’ homology and a 5.11kb 3’ homology fragment was amplified by Pfu PCR from a bacterial artificial chromosome (BAC), which contained the mouse *Gpr55* gene. Both fragments were sub-cloned and sequenced with 3100 BigDye terminator (v3.1 Matrix Standard, Applied Biosystems, Foster city, USA). A region containing the complete *Gpr55* coding sequence (CDS) in exon 2 was replaced with a loxP-flanked Neo cassette (Fig 1A). The targeting vector was linearized and electroporated into embryonic stem (ES) cells (R1/E, Univ. Ave, Toronto, Canada) and selected with G418 (300 μg/ml). ES-cell clones that successfully underwent homologous recombination were identified by PCR screening, and then confirmed by Southern blotting (Fig 1B). Targeted ES-cell clones were injected into C57BL/6 blastocysts, which were subsequently implanted into pseudo pregnant female mice. Chimeric male offspring was bred to C57BL/6 mice to produce germ line transmitted N1 offspring. The Neo cassette was deleted by breeding the N1 mice with Rosa26Cre mice [23], having a pure C57BL/6 background. The *Gpr55* mutant mice were obtained by intercrossing heterozygous mice. Genotyping of the offspring was performed by PCR to detect wild type and mutant alleles by using the following primers: 5′-ATGCGGAATTCCTGTTACCCA -3′ (forward), 5′-CACCCTAGGGCCTCAGTTGTA -3′ (reverse) and 5′-GGAAAGCTGAGATACAGACTT –3′ (reverse). *Gpr55* deficiency was confirmed by RT-PCR analysis. Cerebrums were dissected from 8-week old littermates of *Gpr55* KO homozygous, heterozygous and wild type mice. Reverse transcription was run with SuperScript First-Strand kit (Invitrogen, Carlsbad, USA) followed by a PCR using one primer located in the upstream exon 5'-GGAAAGGGGACTTCTCTTGG-3' and one primer in the deleted region 5'-CACTCCACCAGAGTGCAGAA-3’ (Fig 1C).

### Animal care

All animal experiments were approved by the AstraZeneca internal committee for animal studies as well as the Gothenburg Ethics Committee for Experimental Animals, licence (no: 192–2006) compliant with EU directives on the protection of animals used for scientific purposes.

The animals used in these studies were crossed three generations towards C57Bl/6J (Harlan, the Netherlands). Control animals used throughout the experiments were wild-type (WT) littermates of the *Gpr55* KO mice. All studies were performed on male mice. The mice were housed individually in a temperature controlled room (21°C) with a 12:12 hour light-dark cycle (dawn: 5.30am, lights on: 6.00am, dusk: 5.30pm, lights off: 6pm) and controlled humidity (45–55%). Except where otherwise stated, they had access to a normal chow diet (R36, Lactamin AB, Stockholm, Sweden) and water *ad libitum*. The R36 chow diet contained (weight %): 3.5% cellulose, (energy %): 22.9% protein, 67.1% carbohydrate and 9.6% fat. Environmental enrichment was provided (cartons, wooden tongue depressors & cotton nesting pads) in home cages unless otherwise stated. Upon completion of the studies, mice were fasted for 3h and euthanised by means of cardiac puncture under isoflurane inhalation anaesthesia.

### Comprehensive characterization of *Gpr55* deficient male mice

General phenotypic characterization of the *Gpr55* KO mice followed the protocol previously described [24]. In short, the nose-anus length of mice was measured at 3, 7 and 9 weeks of age and their body weight assessed weekly from 3 to 7 weeks of age, and then in conjunction with *in vivo* experimentation. The condition of fur, nose, eyes and teeth was examined during this initial study period. Normal home cage behaviours, including nesting, grooming, sniffing, rearing and climbing, was monitored by cage-side observations, and basic sensory responses investigated by eye-blink, grip-strength, beam-walking (balance) and click-box (startle reaction) tests during the initial 7 weeks was studied as described [24]. Thermal sensory pain was investigated by the hot water (55°C) immersion tail flick test at 7 weeks of age.

The comprehensive laboratory animal monitoring system (CLAMS, Columbus Instruments, USA) was used to assess energy expenditure (EE), respiratory exchange ratio (RER), spontaneous locomotor activity, as well as diet and water consumption at thermoneutrallity (set for WT mice to be 29.5°C) as previously described [24]. The mice were placed in the CLAMS calorimeter chambers at 9 weeks of age with *ad libitum* access to diet and water for 72 hours. Faeces produced over the experimental time was sampled and energy content determined by a bomb calorimeter (C5000, IKA, Germany).

Rectal core body temperatures were recorded in conscious non-anaesthetised mice in room temperature at day-time (10.00–11.00 am) using a rectal probe.

Body composition was assessed by dual energy X-ray absorptiometry (DEXA, GE Lunar, Madison, USA) in isoflurane anaesthetised mice at 8 weeks of age as previously described [24].

Detailed day-time locomotor activity behaviour was monitored over 1h on two consecutive days in specially designed open field boxes in 10 week old animals, all as previously described [24]. In addition, the mice were assessed in behavioural models commonly used to detect anxiolytic-like (Zero maze system) and antidepressant-like (forced swim test system) drug properties, and a passive avoidance test (Shuttle box system) used to assess memory and learning abilities, according to protocols previously described [24].

### Extended assessments during high fat diet and drug intervention

The *Gpr55* KO and corresponding littermate WT control mice were studied during long-term (28 weeks) exposure to *ad libitum* choice of calorie-dense foodstuff (‘cafeteria diet’: cheese (38% fat, 1% carbohydrate, 20% protein; Arla Ost, Vaästervik, Sweden), milk chocolate (32% fat, 58% carbo- hydrate, 6% protein; Marabou^®^, Kraft Foods, Upplands Vaäsby, Sweden), nougat (39% fat, 47% carbohydrate, 6% protein; Odense Marcipan^®^, Odense, Denmark), and chocolate pastry (31% fat, 52% carbohydrate, and 5% protein; Delicato^®^, Huddinge, Sweden) along with standard R36 chow diet (Lactamin AB, Stockholm, Sweden) to assess any potential interactions of *Gpr55* deficiency with body weight gain and body composition, assessed by DEXA. Furthermore, when in the diet-induced obesity (DIO) condition, *Gpr55* KO and WT littermate control mice were subjected to a 14 days challenge with the CB1 antagonist/GPR55 ligand [8] rimonabant (20 μmol/kg p.o., once daily for 14 days) to examine the relative influence of the CB1 *vs*. GPR55 component in the well-established weight-reducing profile of this agent.

### Statistical analysis

All values are given as group means ± SEM. The *in vivo* phenotyping parameters were compared between WT littermates and *Gpr55* KO mice using Student’s *t*-test, or 2-way ANOVA for repeated measures. A linear regression model for adiposity with body weight and genotype as predictors was used to estimate relative adiposity between genotypes. Probability values p<0.05 were considered statistically significant. The data was log-normalized when appropriate.

## Results

### Generation of *Gpr55* knockout mice

*Gpr55* knock out (*Gpr55* KO) mice were generated by targeted deletion of a 3.1 kb fragment of the coding region in exon 2 (Fig 1A). Mice that were heterozygote for the deletion were intercrossed to produce male homozygote for the deletion (*Gpr55* KO) and wild type (WT) littermate control mice that were studied. Of 67 male pups, 18 were homozygote (27%), 39 were heterozygote (58%) and 10 were WT (15%). Female offspring was not counted or genotyped.

### General characterization

No gross abnormalities were observed at birth, nor during growth, and no significant differences were observed in the condition of fur, nose, eyes and teeth upon ocular inspection comparison of *Gpr55* KO and WT siblings throughout the life-span covering the experiments in the study. In respect to observed behaviours, nesting, grooming, rearing, grid grip strength and climbing demonstrated no differences between *Gpr55* KO and WT control mice (not shown).

### Assessments of behavior and thermal sensory pain

No significant differences were observed between *Gpr55* KO and WT mice in the behavioural anxiolytic/antidepressant-like property assessment models (Zero Maze/Forced Swim Test), nor in the memory/learning ability (Passive Avoidance) paradigm (data not shown).

**Fig 2:**
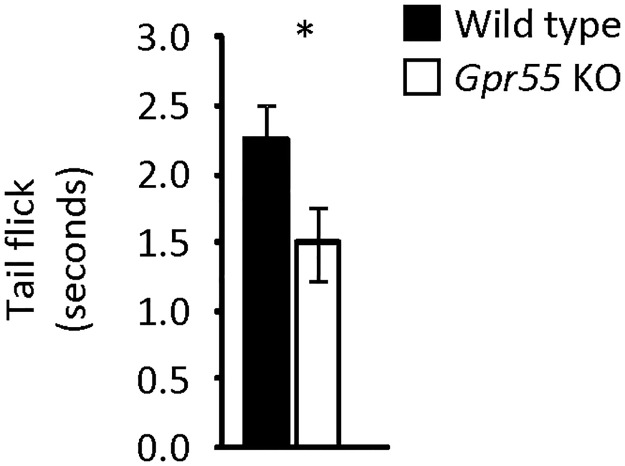
Pain sensitivity. Thermal pain assessed by the tail flick test in *Gpr55* KO (n = 8) and corresponding WT (n = 8) mice. Statistical analysis by Student′s *t*-test. * indicates p<0.05.

*Gpr55* KO mice reacted significantly faster in the hot water immersion tail flick test (Fig 2), indicative of thermal hyperalgesia.

### Body weight, body length and body composition

Although, at 3 weeks of age, the *Gpr55* KO mice tended to be slightly shorter than their WT siblings (WT: 8.29 ± 0.09 cm, *Gpr55* KO: 8.01 ± 0.10 cm; p = 0.06; n = 8 in both groups), no significant differences or tendencies were observed at any of the following assessment time points. In addition, no significant difference was observed regarding body weight gain (3–7 weeks of age). In line with this, neither lean mass, fat mass, nor gross bone mineral density or content (not shown) were significantly altered when *Gpr55* KO and WT mice were compared at 8 weeks of age.

**Fig 3:**
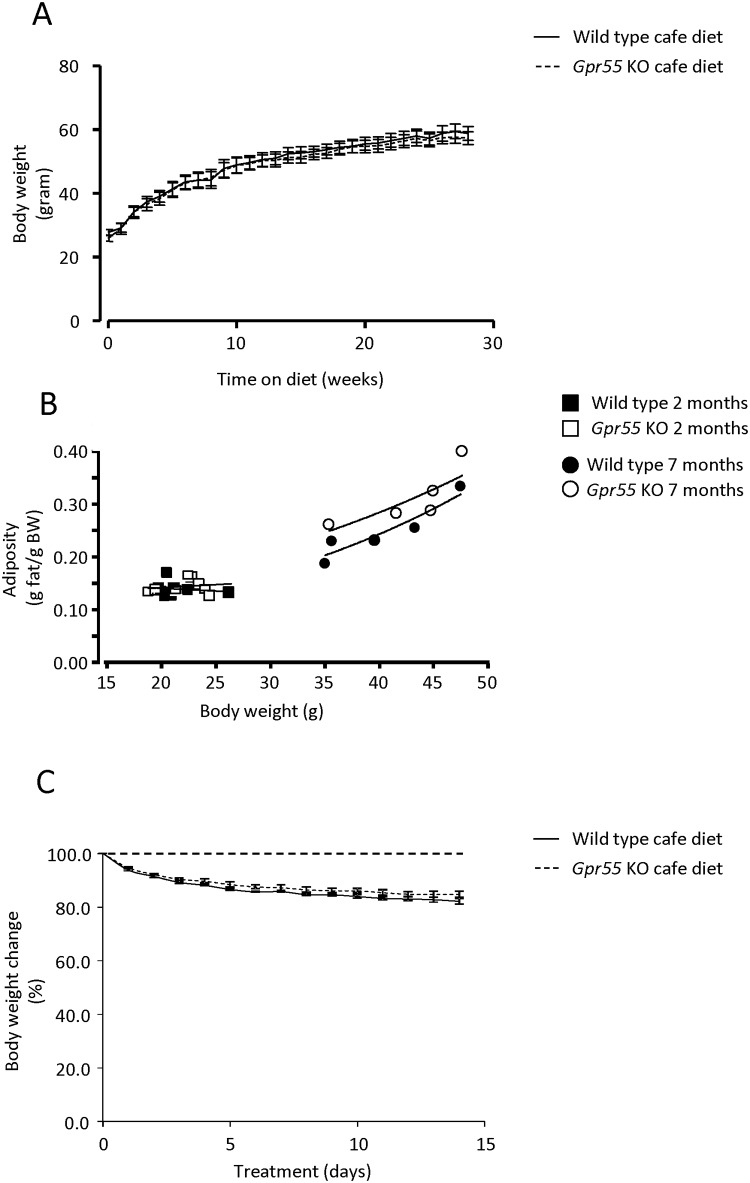
Body weight gain, adiposity and effects of rimonabant. (**A**) Body weight gain over 28 weeks during continuous high fat ‘café diet’ feeding in *Gpr55* KO (n = 5) and corresponding WT (n = 7) mice. Statistical analysis by 2-Way ANOVA for repeated measures, with time and genotype as factors, indicated a significant effect of time (F_28, 280_ = 423.8, p<0.0001) but no difference between genotypes (F_1, 10_ = 0.1030, N.S.). (**B**) Correlation analysis of body weight (g) over relative body fat mass (g fat/ g body weight) in *Gpr55* KO (n = 8) and corresponding WT (n = 8) mice at 2 months of age and in DIO *Gpr55* KO (n = 5) and WT (n = 5) mice at 7 months of age. A linear regression model for adiposity with body weight and genotype as predictors to estimate relative adiposity indicated a non-significant trend (p ~0.10, N.S.) to increased adiposity in DIO *Gpr55* KO *vs*. WT at 7 months of age. (**C**) Effects on body weight of daily dosing of rimonabant (20 μmol/kg p.o., once daily for 14 days) in *Gpr55* KO (n = 5) and corresponding WT (n = 6) mice. Dashed line depicts the (100%) baseline level. Statistical analysis by 2-Way ANOVA for repeated measures, with time and genotype as factors, indicated a significant effect of time (F_14, 126_ = 162.6, p<0.0001) but no difference between genotypes (F_1, 9_ = 0.07729, N.S.).

Consistent with the above, neither was there any significant difference in the rate or extent of body weight gain in *Gpr55* KO mice relative to their WT littermates when fed a calorie-dense cafeteria diet for 28 weeks (Fig 3A, 2-way ANOVA for repeated measures with time and genotype as factors; time, F_28, 280_ = 423.8, p<0.0001; genotype, F_1, 10_ = 0.1030, N.S.; interaction F_28, 280_ = 0.3260, N.S.). Assessment by means of DEXA of body composition after the prolonged cafeteria diet access revealed no significant alterations in any of the parameters assessed, including fat mass, lean mass nor bone mass or bone density. However, a linear regression analysis indicated a slightly though non-significant (p ~ 0.10) increase in adiposity (fat mass relative to body weight) in the old (28 weeks) *Gpr55* KO mice (Fig 3B). Subsequent pharmacological challenge with rimonabant (20 μmol/kg, p.o./q.d.) for 14 days significantly and comparably reduced body weight both in *Gpr55* KO and WT mice by 15–18% from baseline (Fig 3C, 2-way ANOVA for repeated measures with time and genotype as factors; time F_14, 126_ = 162.6, p<0.0001, genotype F_1, 9_ = 0.0773, N.S., interaction F_14, 126_ = 0.2382, N.S.).

### Indirect calorimetry, body temperature and locomotor activity

Indirect calorimetry, diet and water intake as well as day- and night-time locomotor activity was assessed in 9 week old *Gpr55* KO and WT littermate control mice at thermoneutral ambient temperature (29.5°C).

**Fig 4:**
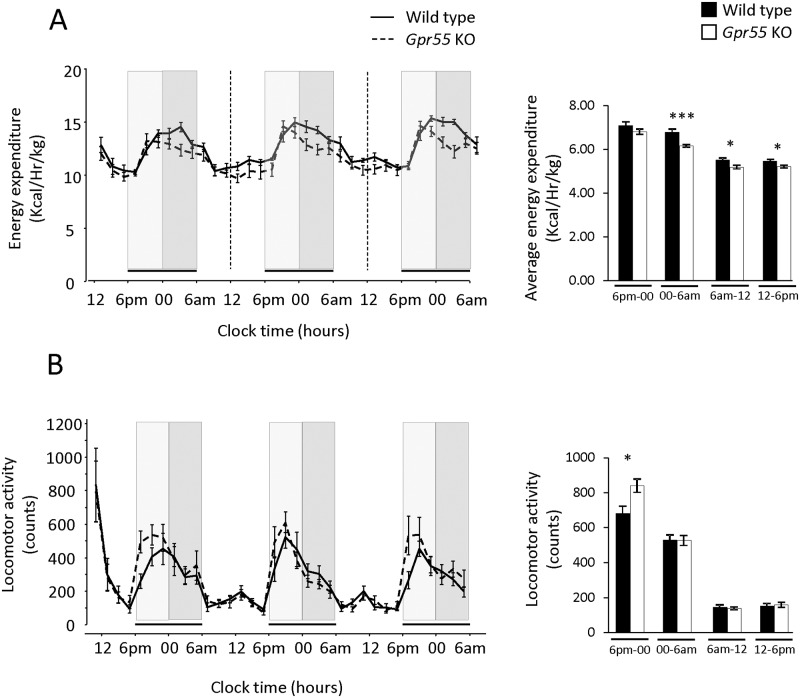
Energy expenditure and locomotor activity. (**A**) Energy expenditure (EE, kcal hr^-1^ kg^-1^) and (**B**) locomotor activity was continuously assessed over 72 hours at a thermoneutral temperature in *Gpr55* KO (n = 8) and corresponding WT (n = 7) mice. Light and dark grey bars indicate initial (6pm– 00) and late (00—6am) part of the night time respectively. Statistical analysis was done by 2-Way ANOVA for repeated measures with time and genotype as factors. Bar charts to the right of the line charts show average night time EE (panel (**A**)) and average night time locomotor activity (panel (**B**)). Statistical comparisons of the averaged data was done by Student′s *t*-test. * p < 0.05; *** p < 0.001.

No significant differences were observed in diet or water consumption or respiratory exchange ratio (RER), indicating unaltered relative usage of energy sources. Energy expenditure (EE, kcal/kg) tended to be lower over the duration of the experimental period for the *Gpr55* KO mice. Although there was no significant difference in EE between *Gpr55* KO and WT mice in the initial part of the night (6.00 pm—midnight) the *Gpr55* KO mice displayed significantly reduced EE during the last 6 hours (midnight—6.00 am) of the dark period as well as during daytime (Fig 4A).

Energy intake and expenditure are typically adjusted to maintain body energy homeostasis. Surprisingly, energy intake or energy uptake (energy intake minus energy left in faeces) was not significantly different between *Gpr55* KO and WT mice indicative of similar energy absorption in these two cohorts. The reduced EE was not correlated to reduced day-time core body temperature, which was not significantly altered in the *Gpr55* KO mice (WT: 37.7 ± 0.2°C; *Gpr55* KO: 37.8 ± 0.2°C).

Paradoxically, locomotor activity was significantly increased during the initial 6 hours of the night period (6.00 pm—midnight) in the *Gpr55* KO mice, when EE was unaltered (Fig 4B). Locomotor activity was not significantly different between the cohorts the latter part of the night (midnight– 6.00 am) when EE was lower in the *Gpr55* KO mice.

**Fig 5:**
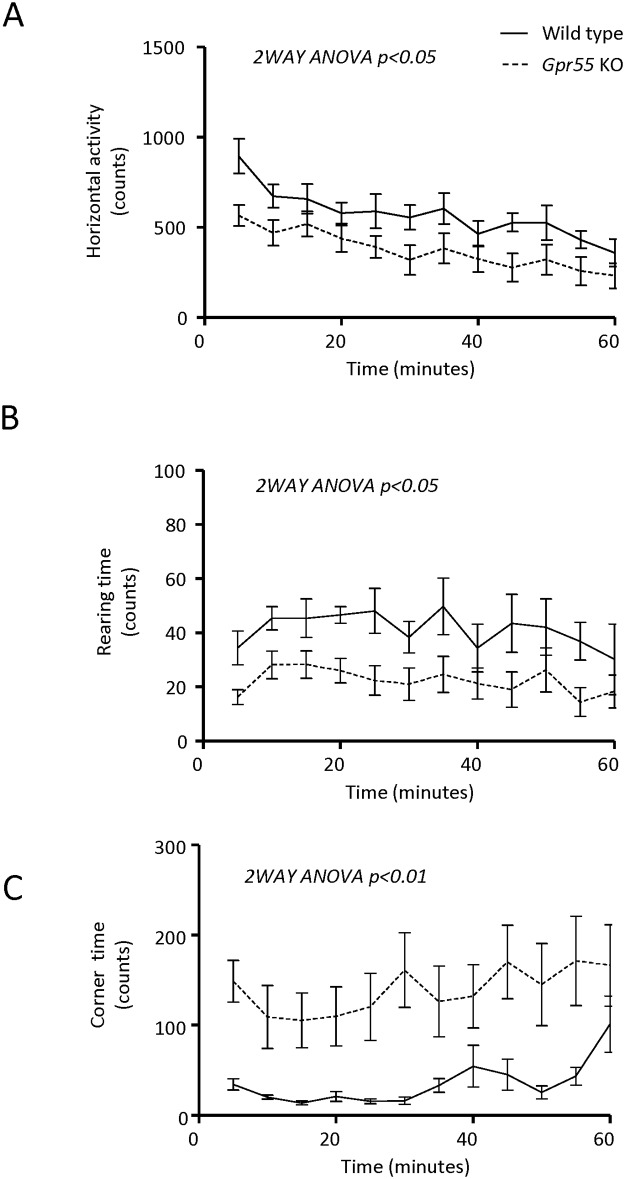
Open Field locomotor activity. (**A**) Horizontal activity, (**B**) rearing time and (**C**) corner time during the second day assessments in *Gpr55* KO (n = 8) and corresponding WT (n = 8) mice. Statistical analysis by 2-Way ANOVA for repeated measures, with time and genotype as factors was carried out for each variable. Horizontal activity: significant effect of time (F_11, 154_ = 13.87, p < 0.0001) and between genotypes (F_1, 14_ = 5.120, p < 0.05) but no interaction (F_11, 154_ = 0.8475, N.S.). Rearing time: significant effect of time (F_11, 154_ = 2.393, p < 0.001) and genotype (F_1, 14_ = 5.543, p < 0.05) but no interaction (F_11, 154_ = 0.51, N.S.). Corner time: significant effect of time (F_11, 154_ = 2.598, p < 0.01) and of genotype (F_11, 154_ = 10.75, p < 0.01) but no interaction (F_11, 154_ = 0.7387, N.S.). Statistically significant differences between *Gpr55* KO and WT are indicated in the Fig.

Locomotor activity was assessed both in conjunction with the indirect calorimetry measurements referred above over 72 continuous hours, as well as in separate 1h-sessions on two consecutive days in an open field activity system during day-time. In this latter model, performance in the arena on the initial experimental day, representing exploratory behaviour, was not significantly different between *Gpr55* KO and WT mice (not shown). During the second day, representing locomotor activity after habituation, the *Gpr55* KO mice displayed low activity and high corner time. In particular, 2-way ANOVA for repeated measures indicated significantly lower horizontal activity (F_1, 14_ = 5.120, p < 0.05) and rearing time (F_1, 14_ = 5.543, p < 0.05) and significantly increased corner time (F_11, 154_ = 10.75, p < 0.01) in the *Gpr55* KO compared to the WT mice (Fig 5A, 5B and 5C). The increased corner time measure in particular is suggestive of more immobility for the *Gpr55* KO compared to WT control mice. For the remainder of the parameters assessed, including peripheral activity and locomotion (sequential beam breaks), the *Gpr55* KO mice appeared consistently less active and less prone to rear. Faeces production over the test period, often used as a measure of stress behaviour and emotionality [25], was not significantly different between *Gpr55* KO and WT mice (not shown).

## Discussion

Many of the CNS actions of both endogenous and exogenous cannabinoid ligands seem to derive from targets other than the commonly and most well documented CB1, CB2 and TRPV1 receptors. In the present paper we describe the phenotypical outcome of a mouse line carrying a disrupted gene encoding one of these alternative cannabinoid targets, *Gpr55*. Similarly to CB1, *Gpr55* mRNA is widely distributed throughout the brain. However, compared to the CB1 receptors, *Gpr55* generally displays lower expression levels, may be more heterogeneously distributed, and the actual CNS levels of receptor protein remains to be verified [26]. This notwithstanding, there is indications in the emerging literature for a possible involvement of GPR55 receptors in non-CB1/non-CB2/non-TRPV1 centrally and peripherally elicited responses to ligands otherwise primarily considered as cannabinoid receptor-active agents.

Since the first publication on GPR55, discussing the possibility that it might belong to the cannabinoid receptor family [8], several studies have been aimed at further understanding the pharmacology of this GPCR (reviewed in [27, 28]). The present report shows that deletion of *Gpr55* produces no conspicuous gross phenotypical, behavioural or pathological changes. Interestingly however, we identified subtle yet significant differences with respect to motor and metabolic parameters between *Gpr55* KO and corresponding WT mice.

Gross behavioural phenotyping showed that *Gpr55* KO mice display similar body weight gain, body composition, energy intake and assimilation, water consumption as WT littermate mice. A slight non-significant shortening of the *Gpr55* KO vs. WT mice was noted only at the 3 week time-point, an observation that may or may not be related to the suggested role of the receptor in bone physiology [21]. No significant differences were observed between the experimental and control cohorts in performance in behavioural tests commonly used to assess anti-anxiety, anti-depressive, cognition or anti-stress properties of drugs. These observations are generally also consistent with a previous study [14] performed in a different strain of *Gpr55* KO mice. That *Gpr55* KO mouse strain was generated by targeting the coding sequence of exon 2 of the *Gpr55* gene and backcrossed to C57BL/6 genetic background, similar to the *Gpr55* KO mouse strain described in the current paper.

### Motor function

The present data and recent observations by others indicate that GPR55 plays a role in the control of central processes involved in behavioural and/or motor function. The data however are partly contradictory, seemingly at least depending on experimental model and conditions used for measurement. Firstly, as a general background, mice are nocturnal, and thus differ in their baseline motor activity across the day-night cycle. We therefore decided to monitor the potential role of GPR55 in two distinctly separate situations: the CLAMS cages (small confined area, thermoneutral temperature, habituation, continuous 72 hrs day & night), and an open field test (large arena, room temperature, 2 days to assess novel and habituated conditions, 1h-sessions during day-time). Assessment of motor activity in 9-week old *Gpr55* KO mice over three consecutive day and night episodes in the CLAMS cages revealed consistently *elevated* horizontal motor activity during the initial 6-hour phase of the dark period. However, during the remainder of the night as well as during daytime, the motor activity did not differ from levels observed in WT mice. In contrast to these findings, the 1-hour open field, day-time light phase activity was indicative of *reductions* in overall mobility in the *Gpr55* KO mice relative to WT littermates. The open field motor assessment is performed in a setting with a much larger arena, has greater resolution allowing for gauging motor activity patterns in a more detailed manner compared to the CLAMS cages, but may additionally involve a stress/emotional component to the response. For comparison, Meadows *et al*. [12] recently reported that 5-months old *Gpr55* KO mice exhibited unaltered forced treadmill activity but *reduced total* 12h-accumulated night-time locomotor activity and voluntary wheel running, although it was not stated if during initial or later parts of the dark phase. Using the same strain of *Gpr55* KO, Wu *et al*. [14] found that while gross motor activity assessed in the open field test was largely unaffected in 2–5 months old mice, a significant impairment was seen with respect to more challenging motor coordination responses, including rotarod and foot-slip test, tentatively in line with the lowered propensity to open field rearing activity observed in the current study. Taken together, the aforementioned motor activity studies thus display overlapping as well as contrasting outcomes. Methodological factors such as the phase of the day-night cycle, the size of the area utilized for assessment, ambient temperature, age, and tentatively also single- *vs*. group-housing of the animals (*e*.*g*., present study *vs*. [14]), may account for some of the discrepancies in this regard. Overall, however, it appears evident that GPR55 demonstrates a subtle but definite modulatory role impacting motor control, and consistent with the presence of GPR55 receptors in brain areas important to motor function (*e*.*g*., [14]). The possibility that GPR55 may play a role in CNS reward systems was also raised in this context by Meadows *et al*. [12], and remains a potential contributory factor to be explored further.

### Pain

While GPR55 does appear to be involved in pain control, available data suggest a clear difference in its impact among different pain modalities (*e*.*g*., mechanical/inflammatory *vs*. thermic; [14, 20]; present data). Our studies showed that *Gpr55* KO mice display a significantly shorter reaction time in respect to the tail-flick test. This is in line with findings by others using the hot-plate model [14, 20], and is indicative of a mild thermic hyperalgesia in the absence of the GPR55 receptor. Generally consistent with a potential analgesic role of GPR55 are also reports that intrathecal administration of AM251 or PEA (a CB1 antagonist and indirect-acting agonist, respectively, which both possess GPR55 agonist activity; *e*.*g*., [8]), results in analgesia in the formalin test [29]. However, in contrast, *Gpr55* KO mice were reported to lack the typical inflammatory mechanical hyperalgesia following intraplantar administration of Freund's complete adjuvant [20]. Clearly, more work is warranted to define the role of GPR55 in relation to type and origin of pain stimulus and the anatomical level(s) involved (peripheral sensory, spinal and/or central).

### Metabolic function

Whether or not GPR55 has a direct role in the development of obesity and diabetes has remained an open question. In two recent review papers, the potential role of GPR55 as a regulator of metabolic function was discussed [11, 22]. While GPR55 is indeed expressed in multiple central and peripheral compartments relevant to energy intake, storage and expenditure (*e*.*g*., hypothalamus, adipose tissue, liver, pancreas), its role and importance in nutritional/energy homeostasis remains unclear due to several contradictory and confusing observations.

It is reported that GPR55 expression in adipose tissue is positively correlated with obesity in humans, particularly in women [30]. It might therefore be expected that deletion of *Gpr55* would yield a lean phenotype. The present data however indicate that the absence of *Gpr55* does not result in any *major* alteration of weight gain or body composition in young or aged male mice, regardless of whether given standard (low-fat) lab chow or being exposed to a protracted high-caloric obesogenic diet. Furthermore, neither was the capacity of the CB1 antagonist/(weak) GPR55 agonist rimonabant (*cf*. [8]) to decrease body weight in the latter diet-induced obese mice significantly different between *Gpr55* KO and WT animals. Thus, while it was proposed that *increased* GPR55 activity might promote obesity in humans [30], genetic deletion of the receptor in male mice does not result in leanness or in attenuated body weight loss by rimonabant treatment (present data). We cannot discard the possibility that rimonabant exposure at the GPR55 receptor was too low under the current drug treatment schedule. Regardless, the findings may tentatively suggest low or no tone at GPR55 under physiological conditions. Needless to say, alternative interpretations relating to species or gender differences and/or compensatory adjustments to genetic *Gpr55* deletion also remain a possible explanation.

Although there was no significant main effect of *Gpr55* deficiency on body weight, body fat mass, calorie intake or assimilation, RER or day time core body temperature, we discovered an intriguing pattern of energy expenditure *vs*. motor activity. These parameters are often linked, but were seemingly dissociated in *Gpr55* KO compared to WT mice in our metabolic cage studies. Thus, during the 6h-time period between lights off and midnight, motor activity was significantly higher in *Gpr55* KO mice without any significant effect on EE. After midnight and throughout the remainder 6h of the night phase, motor activity was normalized but EE was significantly lower in the *Gpr55* KO than in the WT mice. Also during day-time (when rodents are typically at rest) EE was significantly lower in *Gpr55* KO than in WT mice; no differences in motor activity were observed. It would appear that *Gpr55* deficiency is generally associated with low EE, but that its display may be offset in the early part of the dark period by the concomitant increase in motor activity observed in these KO mice. The principal components determining EE are basal metabolism, thermogenesis, thermic effects of diet and physical activity [31]. The low EE in *Gpr55* KO mice is not explained by reduced motor activity in young animals. Altered thermogenesis is also not a likely explanation for low EE as these experiments were conducted under thermoneutrality, and core body temperature was not changed when assessed in room temperature. The reduced EE in the absence of a corresponding effect on energy intake or body weight may indicate increased energy utilization efficiency in the *Gpr55* KO mice. The exact mechanism(s) however remain to be further clarified.

Even if small in size, a persistent lowering of EE will if prolonged be expected to affect energy homeostasis and result in increased body weight. The lack of effect *Gpr55* deficiency on BW and but modest effect on fat mass *vs*. WT mice may therefore seem puzzling. Our data in this regard concur with the observations of Meadows *et al*. [12]. However, in contrast with their report of reduced motor activity in the *Gpr55* KO, we find a transient increase of locomotion during the dark phase (cf. above). In addition to factors already discussed above, age and diet may need to be considered with respect to the net effect of reduced EE on fat mass and body weight. Whereas our metabolic chamber studies were carried out in 9-week old mice, those of the aforementioned authors represent observations in 5-month old mice (and in a different motor activity paradigm). As the animal background strain and the (relatively high-carbohydrate and low-fat) standard lab diets used were similar in the two studies, it may be speculated that an age-related general decay in mobility (cf. [32]) accounts for the somewhat more pronounced—although still modest—adiposity observed in the older mice. For comparison, feeding our *Gpr55* KO mice a high-caloric (high-fat, high-carbohydrate) content diet for 7 months did not influence body weight gain, but showed a small, non-significant trend towards increased adiposity across body weight in the *Gpr55* KO mice *vs*. WT littermates—consistent with the previous report [12]. However, it appears likely that the HFD regimen applied in our studies is close to an obesogenic ‘ceiling’ effect, and therefore that *Gpr55* KO separate only slightly from the WT littermates. Possibly, a more moderate-caloric diet might accentuate the adiposity-promoting action of *Gpr55* deficiency.

The seemingly paradoxical and intriguing pattern of motor activity *vs*. EE and, in turn, body weight/adiposity, may thus be rationalized by sustained actions involving GPR55 on pathways modulating motor as well as energy homeostasis systems. Thus, as motor activity overall declines with increased age it appears reasonable to assume that even the observed subtle reduction in EE may increase fat mass in *Gpr55* deficiency, particularly in conjunction with long-term high-caloric diet exposure. Further detailed studies are required to reconcile these findings, and whether or not they may invoke GPR55-dependent alterations also in, *e*.*g*., circadian behavioral and/or metabolic patterns.

It deserves mention in this context that heterodimeric interactions between CB1 and GPR55 have been described [33]. It has further been suggested that CB_1_ receptor upregulation may trigger a compensatory normalization of food intake following GPR55 deletion, thereby obscuring effects otherwise elicited by a deficiency in the latter [22]. Explanations like these appear, however, less likely to account for the present findings as there was no difference between *Gpr55* KO and WT mice in either the rate or extent of weight gain while on high-caloric diet, nor in weight loss after challenge with the CB_1_ receptor antagonist (/GPR55 agonist; [8]) rimonabant (Fig 4).

## Concluding remarks

The present study has pinpointed some of the subtle phenotypical traits in the mouse following genetic deletion of *Gpr55*. The data demonstrate clearly discernible alterations in motor, pain and metabolic variables. Several effects of cannabinoid-receptor active agents have defied full identification within a CB1 and/or CB2 receptor framework (*cf*. [27, 28]). As some such endogenous and exogenous CB receptor ligands are additionally known to interact with GPR55 sites in an agonist or antagonist capacity (reviewed by [11, 22]), it remains to be determined to what extent GPR55 may be involved in mediation of non-CB1/CB2 actions of these agents. Taken together, the modest (and temporally transient) magnitude of the effects seen in the *Gpr55* deficient mice would appear to be in general agreement with the idea that GPR55 serves a modulatory role jointly with CB1 and/or CB2 receptors in central and/or peripheral control of motor, pain and metabolic function (*cf*., *e*.*g*., [26, 34]). It might be speculated that approaches involving more drastic experimental challenges would be required to reveal enhanced insight into the function of GPR55. Further studies are clearly required to unveil physiological/pathophysiological states in which GPR55 enhancement or blockade might become therapeutically exploitable.

## References

[1] Kogan NM, Mechoulam R. Cannabinoids in health and disease. Dialogues Clin Neurosci. 2007;9(4):413–30. 18286801 10.31887/DCNS.2007.9.4/nkogan PMC3202504

[2] Devane WA, Dysarz FA 3rd, Johnson MR, Melvin LS, Howlett AC. Determination and characterization of a cannabinoid receptor in rat brain. Mol Pharmacol. 1988;34(5):605–13. 2848184

[3] Matsuda LA, Lolait SJ, Brownstein MJ, Young AC, Bonner TI. Structure of a cannabinoid receptor and functional expression of the cloned cDNA. Nature. 1990;346(6284):561–4. 10.1038/346561a0 2165569

[4] Munro S, Thomas KL, Abu-Shaar M. Molecular characterization of a peripheral receptor for cannabinoids. Nature. 1993;365(6441):61–5. 10.1038/365061a0 7689702

[5] Caterina MJ, Schumacher MA, Tominaga M, Rosen TA, Levine JD, Julius D. The capsaicin receptor: a heat-activated ion channel in the pain pathway. Nature. 1997;389(6653):816–24. 10.1038/39807 9349813

[6] Gantz I, Muraoka A, Yang YK, Samuelson LC, Zimmerman EM, Cook H, et al Cloning and chromosomal localization of a gene (GPR18) encoding a novel seven transmembrane receptor highly expressed in spleen and testis. Genomics. 1997;42(3):462–6. 10.1006/geno.1997.4752 9205118

[7] Moran BM, Flatt PR, McKillop AM. G protein-coupled receptors: signalling and regulation by lipid agonists for improved glucose homoeostasis. Acta Diabetol. 2016;53(2):177–88. 10.1007/s00592-015-0826-9 26739335

[8] Ryberg E, Larsson N, Sjogren S, Hjorth S, Hermansson NO, Leonova J, et al The orphan receptor GPR55 is a novel cannabinoid receptor. Br J Pharmacol. 2007;152(7):1092–101. 10.1038/sj.bjp.0707460 17876302 PMC2095107

[9] Imbernon M, Whyte L, Diaz-Arteaga A, Russell WR, Moreno NR, Vazquez MJ, et al Regulation of GPR55 in rat white adipose tissue and serum LPI by nutritional status, gestation, gender and pituitary factors. Mol Cell Endocrinol. 2014;383(1–2):159–69. 10.1016/j.mce.2013.12.011 24378736

[10] Sawzdargo M, Nguyen T, Lee DK, Lynch KR, Cheng R, Heng HH, et al Identification and cloning of three novel human G protein-coupled receptor genes GPR52, PsiGPR53 and GPR55: GPR55 is extensively expressed in human brain. Brain Res Mol Brain Res. 1999;64(2):193–8. 9931487 10.1016/s0169-328x(98)00277-0

[11] Simcocks AC, O'Keefe L, Jenkin KA, Mathai ML, Hryciw DH, McAinch AJ. A potential role for GPR55 in the regulation of energy homeostasis. Drug Discov Today. 2014;19(8):1145–51. 10.1016/j.drudis.2013.12.005 24370891

[12] Meadows A, Lee JH, Wu CS, Wei Q, Pradhan G, Yafi M, et al Deletion of G-protein-coupled receptor 55 promotes obesity by reducing physical activity. Int J Obes (Lond). 2016;40(3):417–24. 26447738 10.1038/ijo.2015.209

[13] Shore DM, Reggio PH. The therapeutic potential of orphan GPCRs, GPR35 and GPR55. Front Pharmacol. 2015;6:69 10.3389/fphar.2015.00069 25926795 PMC4397721

[14] Wu CS, Chen H, Sun H, Zhu J, Jew CP, Wager-Miller J, et al GPR55, a G-protein coupled receptor for lysophosphatidylinositol, plays a role in motor coordination. PLoS One. 2013;8(4):e60314 10.1371/journal.pone.0060314 23565223 PMC3614963

[15] Oka S, Nakajima K, Yamashita A, Kishimoto S, Sugiura T. Identification of GPR55 as a lysophosphatidylinositol receptor. Biochem Biophys Res Commun. 2007;362(4):928–34. 10.1016/j.bbrc.2007.08.078 17765871

[16] Pertwee RG. Receptors and channels targeted by synthetic cannabinoid receptor agonists and antagonists. Curr Med Chem. 2010;17(14):1360–81. 20166927 10.2174/092986710790980050 PMC3013229

[17] Pertwee RG, Howlett AC, Abood ME, Alexander SP, Di Marzo V, Elphick MR, et al International Union of Basic and Clinical Pharmacology. LXXIX. Cannabinoid receptors and their ligands: beyond CB(1) and CB(2). Pharmacol Rev. 2010;62(4):588–631. 10.1124/pr.110.003004 21079038 PMC2993256

[18] Perez-Gomez E, Andradas C, Flores JM, Quintanilla M, Paramio JM, Guzman M, et al The orphan receptor GPR55 drives skin carcinogenesis and is upregulated in human squamous cell carcinomas. Oncogene. 2013;32(20):2534–42. 10.1038/onc.2012.278 22751111

[19] Romero-Zerbo SY, Rafacho A, Diaz-Arteaga A, Suarez J, Quesada I, Imbernon M, et al A role for the putative cannabinoid receptor GPR55 in the islets of Langerhans. J Endocrinol. 2011;211(2):177–85. 10.1530/JOE-11-0166 21885477

[20] Staton PC, Hatcher JP, Walker DJ, Morrison AD, Shapland EM, Hughes JP, et al The putative cannabinoid receptor GPR55 plays a role in mechanical hyperalgesia associated with inflammatory and neuropathic pain. Pain. 2008;139(1):225–36. 10.1016/j.pain.2008.04.006 18502582

[21] Whyte LS, Ryberg E, Sims NA, Ridge SA, Mackie K, Greasley PJ, et al The putative cannabinoid receptor GPR55 affects osteoclast function in vitro and bone mass in vivo. Proc Natl Acad Sci U S A. 2009;106(38):16511–6. 10.1073/pnas.0902743106 19805329 PMC2737440

[22] Liu B, Song S, Jones PM, Persaud SJ. GPR55: from orphan to metabolic regulator? Pharmacol Ther. 2015;145:35–42. 10.1016/j.pharmthera.2014.06.007 24972076

[23] Soriano P. Generalized lacZ expression with the ROSA26 Cre reporter strain. Nat Genet. 1999;21(1):70–1. 10.1038/5007 9916792

[24] Gerdin AK, Surve VV, Jonsson M, Bjursell M, Bjorkman M, Edenro A, et al Phenotypic screening of hepatocyte nuclear factor (HNF) 4-gamma receptor knockout mice. Biochem Biophys Res Commun. 2006;349(2):825–32. 10.1016/j.bbrc.2006.08.103 16945327

[25] Voikar V, Vasar E, Rauvala H. Behavioral alterations induced by repeated testing in C57BL/6J and 129S2/Sv mice: implications for phenotyping screens. Genes Brain Behav. 2004;3(1):27–38. 14960013 10.1046/j.1601-183x.2003.0044.x

[26] Balenga NA, Henstridge CM, Kargl J, Waldhoer M. Pharmacology, signaling and physiological relevance of the G protein-coupled receptor 55. Adv Pharmacol. 2011;62:251–77. 10.1016/B978-0-12-385952-5.00004-X 21907912

[27] Ross RA. The enigmatic pharmacology of GPR55. Trends Pharmacol Sci. 2009;30(3):156–63. 10.1016/j.tips.2008.12.004 19233486

[28] Sharir H, Abood ME. Pharmacological characterization of GPR55, a putative cannabinoid receptor. Pharmacol Ther. 2010;126(3):301–13. 10.1016/j.pharmthera.2010.02.004 20298715 PMC2874616

[29] Naderi N, Majidi M, Mousavi Z, Khoramian Tusi S, Mansouri Z, Khodagholi F. The interaction between intrathecal administration of low doses of palmitoylethanolamide and AM251 in formalin-induced pain related behavior and spinal cord IL1-beta expression in rats. Neurochem Res. 2012;37(4):778–85. 10.1007/s11064-011-0672-2 22201038

[30] Moreno-Navarrete JM, Catalan V, Whyte L, Diaz-Arteaga A, Vazquez-Martinez R, Rotellar F, et al The L-alpha-lysophosphatidylinositol/GPR55 system and its potential role in human obesity. Diabetes. 2012;61(2):281–91. 10.2337/db11-0649 22179809 PMC3266411

[31] Bjursell M, Gerdin AK, Lelliott CJ, Egecioglu E, Elmgren A, Tornell J, et al Acutely reduced locomotor activity is a major contributor to Western diet-induced obesity in mice. Am J Physiol Endocrinol Metab. 2008;294(2):E251–60. 10.1152/ajpendo.00401.2007 18029443

[32] Shoji H, Takao K, Hattori S, Miyakawa T. Age-related changes in behavior in C57BL/6J mice from young adulthood to middle age. Mol Brain. 2016;9:11 10.1186/s13041-016-0191-9 26822304 PMC4730600

[33] Kargl J, Balenga N, Parzmair GP, Brown AJ, Heinemann A, Waldhoer M. The cannabinoid receptor CB1 modulates the signaling properties of the lysophosphatidylinositol receptor GPR55. J Biol Chem. 2012;287(53):44234–48. 10.1074/jbc.M112.364109 23161546 PMC3531739

[34] Balenga NA, Martinez-Pinilla E, Kargl J, Schroder R, Peinhaupt M, Platzer W, et al Heteromerization of GPR55 and cannabinoid CB2 receptors modulates signalling. Br J Pharmacol. 2014;171(23):5387–406. 10.1111/bph.12850 25048571 PMC4294047

